# Curves of the Spine, etc.

**Published:** 1889-02-16

**Authors:** 


### Surgery and Therapeutics.

### CURVES OF THE SPINE, ETC.

Mr. T. W. Nunn, consulting surgeon to the Middlesex Hospital, has published a pamphlet, read before the Staffordshire Branch of the British Medical Association last year, on "Certain Disregarded Effects of Development, Chiefly in Relation to the Curves of the Spine." The spinal column in early life is nearly straight, but as development proceeds, the spine assumes certain curves. It bulges inwards about the neck and outwards about the chest, inwards again below, and outwards again towards the extremity, the very lowest part again curving inwards. Defective development may, the author states, be positive or negative, that is, may consist in the non-growth of what ought to grow, or in the persistence of a condition which naturally is but temporary. Whatever form of defective development presents, the results may be direct and mechanical, or reflex. Mr. Nunn has observed that certain acquired distortions, especially of the lower limbs, are associated with abnormalities of the antero-posterior curves of the spine. The whole of a limb or groups of muscles may be affected, or single muscles, or even ligaments or bones. An inequality in the length of the lower limbs is also another defect. An abnormality which belongs to the class of imperfect developments is an absence or a lessening of the proper backward spinal curvature, the spine retaining its infant perpendicularity. This causes the shoulder-blades to appear to project, and the want of curve diminishes the capacity of the chest, and interferes with the action of the heart. It is well known that by reflex nervous action various parts of the body become diseased, as a consequence of some other distant organ being affected. For example, bad teeth may cause disease in the eye, convulsions, or St. Vitus's dance. It is not, therefore, surprising that when the spine is in any way not perfectly natural results may occur in any part of the body; for it is from the spine that many nerves issue. There is also, as previously referred to, the mechanical effect of alterations in the form of the spine. Mr. Nunn has done good service in calling attention to these hitherto disregarded effects of development of the curves of the spinal column.

